# Performance-informed EEG analysis reveals mixed evidence for EEG signatures unique to the processing of time

**DOI:** 10.1007/s00426-018-1039-y

**Published:** 2018-06-20

**Authors:** Nadine Schlichting, Ritske de Jong, Hedderik van Rijn

**Affiliations:** 1 Department of Experimental Psychology, University of Groningen, Groningen, The Netherlands; 2 Research School of Behavioural and Cognitive Neurosciences, University of Groningen, Groningen, The Netherlands

## Abstract

Certain EEG components (e.g., the contingent negative variation, CNV, or beta oscillations) have been linked to the perception of temporal magnitudes specifically. However, it is as of yet unclear whether these EEG components are really unique to time perception or reflect the perception of magnitudes in general. In the current study we recorded EEG while participants had to make judgments about duration (*time* condition) or numerosity (*number* condition) in a comparison task. This design allowed us to directly compare EEG signals between the processing of time and number. Stimuli consisted of a series of blue dots appearing and disappearing dynamically on a black screen. Each stimulus was characterized by its duration and the total number of dots that it consisted of. Because it is known that tasks like these elicit perceptual interference effects that we used a maximum-likelihood estimation (MLE) procedure to determine, for each participant and dimension separately, to what extent time and numerosity information were taken into account when making a judgement in an extensive post hoc analysis. This approach enabled us to capture individual differences in behavioral performance and, based on the MLE estimates, to select a subset of participants who suppressed task-irrelevant information. Even for this subset of participants, who showed no or only small interference effects and thus were thought to truly process temporal information in the time condition and numerosity information in the number condition, we found CNV patterns in the time-domain EEG signals for both tasks that was more pronounced in the time-task. We found no substantial evidence for differences between the processing of temporal and numerical information in the time–frequency domain.

## Introduction^1^

Studies investigating the neural processes underlying the perception of time in humans have suggested that there are activation patterns and neural mechanisms that are unique to timing. One neural activation pattern that has been associated with the perception and production of intervals in the order of hundreds of milliseconds to multiple seconds is a slow negative deflection measured using EEG at fronto-central and parietal–central locations. The association is driven by the observation that amplitude variations of this slow contingent negative variation (CNV) are related to variations in temporal performance, that is, subjective timing (Bendixen, Grimm, & Schröger, 2005; Durstewitz, 2004; Macar, & Vidal, 2004; Macar, Vidal, & Casini, 1999; Pfeuty, Ragot, & Pouthas, 2005). Critically, it is assumed that the CNV reflects the accrual of temporal information over time, the core component of the clock- or pacemaker-based theories of interval timing (see for a discussion of these models, van Rijn, Gu, & Meck, 2014). However, failures to replicate performance-dependent variations in CNV amplitudes (Kononowicz, & van Rijn, 2011) and results that are difficult to align with the view that the CNV represents the core component of timing-tasks (Ng, & Penney, 2014), have led to a re-evaluation of the role of processes reflected by the CNV. This re-evaluation is further supported by the observation that other EEG components than the CNV track subjective timing more accurately than the CNV, and that these components even correlate with subjective timing when no CNV is present (Kononowicz, & van Rijn, 2014a).

In earlier work, we have argued (Kononowicz, & Penney, 2016; Kononowicz, & van Rijn, 2011; van Rijn, Kononowicz, Meck, Ng, & Penney, 2011; see also Ng, & Penney, 2014) that amplitude variations might reflect more general processes that are necessary for any timing task (e.g., the setting of decision thresholds, Boehm, van Maanen, Forstmann, & van Rijn, 2014), but not the temporal accumulation process as such. Interestingly, this convolution of pure timing and the auxiliary processes required to perform a timing task has been acknowledged in fMRI studies aimed at unraveling the neural foundations of interval timing. In most fMRI experimental designs, neural activity measured during a timing task is compared to the activity elicited by a control task that does not have a temporal component, but is otherwise as similar as possible (and see Kulashekhar, Pekkola, Palva, & Palva, 2016, for an MEG study using a similar setup as the current study). This can be conceptualized as interpreting the differences in activation between both tasks as the reflection of pure timing components. Examples of control tasks are typically tasks in which the magnitude of another dimension needs to be evaluated; for example, color (Bueti, & Macaluso, 2011; Coull, Vidal, Nazarian, & Macar, 2004) or space (Coull, Charras, Donadieu, Droit-Volet, & Vidal, 2015). Based on such fMRI studies, widespread brain networks linked specifically to the processing of temporal magnitudes have been identified. Among these, the supplementary motor area (SMA) has been suggested as a key component in interval timing (Coull, Vidal, & Burle, 2016; Wiener, Turkeltaub, & Coslett, 2010). For example, Coull et al. (2015) showed that SMA activity increases incrementally with increasing stimulus duration. In their experiment, participants had to estimate either the duration or distance of the trajectory of a moving dot. The contrast between duration and distance conditions showed that SMA was activated only during the temporal task, and, furthermore, activity in this region was positively correlated with stimulus duration, but not distance. Like the earlier discussed CNV results, these results were interpreted to mean that the SMA functions as an active accumulator of temporal information.

**Fig. 1:**
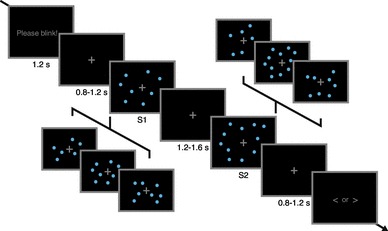
Experimental design. In this classical comparison task, participants had to judge whether the second stimulus was longer or shorter than the first stimulus (*time* dimension), or consisted of fewer or more dots (*number* dimension). Participants were cued before sub-blocks of eight trials which dimension would be the target dimension for the next trials. Stimuli consisted of clouds of small blue dots which appeared and disappeared dynamically on the screen. Single trials started with a “Please blink!” instruction to reduce eye movement artifacts during stimulus presentation. Either S1 or S2 was always the standard stimulus, lasting for 1.8 s and consisting of 30 dots in total, while the other stimulus could take on one of six comparison magnitudes in both dimensions

As mentioned, the use of comparison tasks is rarely utilized in EEG or MEG studies, rendering it possible that observed differences are not due to differences in timing, but rather due to differences in auxiliary processes that correlate with the length of the perceived intervals (e.g., changes in response caution due to the changes in hazard rate). In the current study, we utilized a comparison task to investigate differences in EEG patterns between timing and non-timing tasks that share most other properties. Participants were asked to compare two sequentially presented durations and indicate whether the second duration was longer or shorter than the first. The durations were presented as dynamic displays of blue dots appearing and disappearing on a black screen, together forming a cloud of dots (see Fig. 1; Lambrechts, Walsh, & Van Wassenhove, 2013, for a similar task design). Each stimulus was characterized by its duration and the total number of dots which it contained. In each trial, either the first or the second stimulus was always the standard stimulus (i.e., lasting for the standard duration and containing the standard number of dots), while the other stimulus could take on one of six comparison intervals/number of dots. In half of the trials, participants were asked to make judgements on numerosity for the first and second stimulus instead of the temporal-judgement task. Crucially, the same stimuli were used in both tasks to match for task difficulty, accumulative nature, sustained attention to the stimuli, and working memory demands. Furthermore, non-timing- and non-numerosity-related cognitive processes (e.g., decision-making or preparation of motor responses) are assumed to be similar in both conditions.

This paradigm will allow us to assess whether any observed CNV differences are unique to timing or whether they are shared by both tasks and thus represent more general processes. Apart from assessing the contribution of the CNV, a time-domain signal, to timing-specific processes, this setup also allows for determining the contribution of signals in the time–frequency domain. This is specifically relevant as recent explorations of oscillatory activity in the frequency domain in timing-tasks have suggested that timing is associated with activity in different frequency bands (Kononowicz, & van Wassenhove, 2016; Wiener, & Kanai, 2016). Frequency bands that have been associated with interval timing or time-dependent tasks are theta–power (temporal-order maintenance in working memory: Hsieh, Ekstrom, & Ranganath, 2011; Roberts, Hsieh, & Ranganath, 2013), alpha-power and -phase (temporal prediction: Rohenkohl, & Nobre, 2011; Samaha, Bauer, Cimaroli, & Postle, 2015); (duration maintenance in working memory: Chen, Chen, Kuang, & Huang, 2015), and beta-power (beta oscillations are correlated with duration estimates: Kulashekhar et al., 2016; beta-power measured at the onset of an interval production predicts produced duration: Arnal, Doelling, & Poeppel, 2015; Kononowicz, & van Rijn, 2015; Kononowicz, & van Rijn, 2014b). Yet, as for the time-domain studies discussed above, no control condition was present to distinguish pure timing signals from auxiliary processes.

To summarize, here, we will compare differences observed in EEG voltage (i.e., in the time-domain) and EEG power (i.e., in the time–frequency domain) between the processing of temporal and numerical information to reveal which EEG components are unique to time processing. As the processing of temporal and numerical information is based on identical stimuli, with similar instructions, any observed differences between both conditions are attributable to the differences between time and number processing, elucidating the components that are specific to the processing of time.

## Materials and methods

### Participants

For the initial sample 27 healthy participants with normal or corrected-to-normal vision were recruited. They received partial course credits or a financial compensation of 15 euros for their participation. Informed consent as approved by the Ethical Committee Psychology of the University of Groningen (identification number 15104-NE) was obtained before testing. The data of five participants were not included in the analysis because of excessive artifacts in over 30% of the trials. Because of creating subgroups of participants in the post hoc analysis, we extended the sample by 28 participants, of which six were excluded from the analysis because of artifacts. The final sample comprised data of 44 participants (38 right-handed and 29 female) aged between 18 and 29 years (*M* = 21.77 years).

### Stimuli and experimental design

Clouds of dynamically appearing and disappearing blue dots presented within a circular area around the fixation cross served as stimuli. The duration of each stimulus was marked by the appearance of the first dots (onset) and disappearance of the last dots (offset). The number of dots was determined by the total number of unique dots presented. Each stimulus could vary simultaneously and independently in duration, referred to as *time*, and in the *number* of dots displayed during the duration. We chose to present the numerosity dimension dynamically over time to equate the two tasks as much as possible—including the accumulative nature timing-tasks inherently entail (Coull et al., 2015; for similar task designs, see Lambrechts et al., 2013; Martin, Wiener, & van Wassenhove, 2017).

In a comparison, task participants had to judge whether the second stimulus (S2) presented in a trial was shorter or longer (*time* dimension) or consisted of more or fewer dots (*number* dimension) than the first stimulus (S1), whereby either S1 or S2 was always the standard stimulus. Participants were cued at the start of each sub-block of 8 trials whether they had to make judgements on *time* or on *number* throughout that sub-block. Figure 1 shows a visual depiction of an experimental trial; in addition, a video demonstration can be found online at osf.io/usjh4.

The lifetime of each dot (i.e., the interval between appearance and disappearance of the dot) was sampled from a uniform distribution between 0.4 and 0.8 s. Multiple dots could be visible at the same time, and it was ensured that at least one dot would be on screen at any moment during the interval. Dots had a size of 0.1 degree of visual angle (5 px), and appeared within a virtual ring with an outer radius of 2.8 (150 px) and an inner radius of 0.9 (50 px) degree of visual angle around the fixation cross. Positions of single dots within one trial were chosen randomly, with the constraint that dots could not overlap in space (i.e., they were separated by at least 0.2 degree of visual angle (10 px)). The experiment was run in Matlab 7.13 (The MathWorks) using the Psychophysics toolbox version 3.0.12 (Brainard, 1997) in Windows 7 (version 6.1).

The standard stimulus (T_S_N_S_) lasted 1.8 s and consisted of 30 dots. Thus, the standard stimulus was always T_S_N_S_ in both *time-* and *number*-trials. The probe stimuli in both dimensions took six possible magnitude values defined as 1.1^−4^, 1.1^−2^, 1.1^−1^, 1.1^1^, 1.1^2^, and 1.1^4^ times the standard magnitude^2^ (hereafter referred to as T_1_, T_2_, T_3_, T_4_, T_5_, and T_6_ for *time* magnitudes, and N_1_, N_2_, N_3_, N_4_, N_5_, and N_6_ for *number* magnitudes). Probe stimuli can be further categorized as congruent (i.e., both dimensions vary in the same direction, e.g., shorter and fewer dots as in stimulus T_1_N_2_) and incongruent (i.e., dimensions vary in different directions, e.g., shorter and more dots as in stimulus T_1_N_4_).

It would seem natural to independently select the duration and the number of dots of non-standard stimuli. This would be an appropriate procedure for static stimuli, but, for the dynamic stimuli used in this experiment, such a procedure would generate large fluctuations across non-standard stimuli in the average rate of drop appearance/disappearance, which corresponds closely to the average number of actually visible drops at any moment. More importantly, such fluctuations in this salient emergent feature would be strongly correlated with fluctuations in both duration (*r* = − .66) and number of drops (*r* = .70). As a consequence, participants might opt to base their judgments, in both the time-task and the number-task, on the average rate of drop appearance instead of on the cued dimension, and still perform quite accurately. This potential problem can be effectively addressed only by allowing some degree of positive dependence between duration and number of dots in constructing non-standard stimuli—that is, some compromise is required to balance the two mutually incompatible desiderata of low correlations between average rate and time and number on one hand, and a low correlation between time and number on the other. Such a compromise was achieved by conditional constrained random sampling of the uncued magnitude of the non-standard stimulus. Specifically, the uncued magnitude was chosen randomly from a weighted uniform distribution. Weights that we finally decided on were 0.8 for the same magnitude as the cued magnitude, and 0.75, 0.55, 0.25, 0.05, and 0 for magnitudes with increasing distance from the cued magnitude (hence, T_1_N_6_ or T_6_N_1_ did not occur in the experiment). Using these weights, we simulated 10,000 stimuli and found a correlation between time and number of *r* = .51, and a correlation of *r* = .50 between number (*r* = − .47 for time) and rate of drop appearance. We deemed this compromise acceptable, as these unavoidable correlations would seem sufficiently small to ensure that average accuracy would be sufficiently compromised if judgments would be based on the uncued dimension or on average rate instead of on the cued dimension. The script running this simulation and additional ones exploring different ways to combine cued and uncued magnitudes can be found online at osf.io/usjh4.

### Procedure

Electroencephalograms were recorded, while participants were comfortably seated with their heads positioned on a chin rest. Stimuli were displayed on a 1280 × 1024 LED-based monitor screen (Iiyama ProLite G2773HS) with a refresh rate of 100 Hz. Participants were seated approximately 100 cm away from the display.

The experiment was divided into four blocks; each block consisting of 80 trials. Within each block, *time-* and *number*-trials were alternating in sub-blocks of eight trials each. The order of these sub-blocks was counterbalanced between participants. Before each sub-block, participants were cued whether they had to make judgements on *time* or on *number*. In each block, in half of the *time-*trials, T_S_ was presented first (i.e., as S1); in the other half, T_S_ was presented second (i.e., as S2). The order of trials was randomized. The probe stimulus in each of the two conditions (T_S_ as S1 and T_S_ as S2) was longer than T_S_ in half of the trials (T_4_–T_6_), and shorter in the other half (T_1_–T_3_). Out of the 40 *time*-trials, T_2_–T_5_ appeared eight times each as the probe stimulus; T_1_ and T_6_ appeared four times each. The same distribution held for *number*-trials.

Figure 1 shows a visual depiction of an experimental trial. Each trial started with a “Please blink!” instruction displayed for 1.2 s, followed by the presentation of a grey fixation cross for a duration sampled from a uniform distribution between 0.8 and 1.2 s. Then, S1 and S2 were presented consecutively with an inter-stimulus-interval sampled from a uniform distribution between 1.2 and 1.6 s. The fixation cross remained on screen for another uniformly sampled 0.8–1.2 s before the response screen appeared and stayed until a response was given. Participants were instructed to press *S* on a conventional US-Qwerty keyboard if they perceived S2 as shorter or consisting of fewer dots than S1, and *L* if they perceived S2 as longer or consisting of more dots than S1. A blank screen appeared for a uniformed sampled 0.8–1.2 s before the next trial started.

### Behavioral data analysis

Proportions of “longer”/“more” responses were computed for each participant, dimension, and magnitude separately. For each participant, data were then fitted to a logistic function for the two dimensions separately using the Psignifit toolbox version 3.0 (Fründ, Haenel, & Wichmann, 2011) in Matlab 8.5. As a measure of response accuracy, we computed the Weber Ratio (WR) from the logistic functions. The Weber Ratio was computed as half the distance between values that support 25 and 75% of “longer” (“more”) responses normalized by the Point of Subjective Equality following Lambrechts et al. (2013). A WR closer to 0 indicates higher response accuracy. To test whether the *time*- and *number*-task were equated in difficulty, paired-sample *t* tests comparing WRs in the two dimensions were performed. For all results, we calculated Bayes Factors to quantify the evidence in favor of the null hypothesis using the *ttestBF* function from the *BayesFactor* package in R (Morey, Rouder, & Jamil, 2014) using the default (Cauchy) prior scaling of $\sqrt{2}/2$.The evidence for H_0_ over H_1_ will be denoted as BF_01_.

### EEG data acquisition and preprocessing

EEG signals were recorded from 30 Ag/AgCl electrodes placed at AFz, F3, F1, Fz, F2, F4, FC5, FC3, FC1, FCz, FC2, FC4, FC6, C5, C3, C1, Cz, C2, C4, C6, CP3, CP1, CPz, CP2, CP4, P3, Pz, P4, O1, and O2 (WaveGuard EEG cap, eemagine Medical Imaging Solutions GmbH, Berlin, Germany), POz (ground electrode), and additionally from left and right mastoids. Online reference was set to the average of all 30 electrodes. The sampling rate was 500 Hz (TMS International, no online filters, impedances kept below 10 kΩ). The electrooculogram was recorded from vertical and horizontal bipolar montages to measure blinks and eye movements.

Offline data analysis was performed using FieldTrip (version 20160727; Oostenveld, Fries, Maris, & Schoffelen, 2011) and customized Matlab scripts. EEG recordings were rereferenced to the averaged mastoids; bandpass filtered between 0.01 and 80 Hz using a Butterworth IIR filter. Epoched data (− 0.8 to 2.4 s, time-locked to the onset of the standard stimulus) were corrected for artifacts (eye movements, noisy channels) using independent component analysis. Subsequently, epochs containing a signal range larger than 120 µV in any EEG channel were automatically detected and excluded from further analysis (on average 9.84% (95% CI [8.32 11.35]) of all 320 trials were discarded) and data were downsampled to 250 Hz. For the CNV analysis, epochs were additionally low-pass filtered at 5 Hz using the default filter settings in FieldTrip, and the average voltage over 0.2 s prior to stimulus onset was used for baseline correction.

To examine oscillatory responses, full stimulus epochs were analyzed in the time–frequency domain. Single-trial time-domain trials were submitted to a time–frequency analysis based on multitapers. Here, we used Hanning tapers with a time resolution of 0.01 s, frequencies of interest were set between 2 and 30 Hz in steps of 0.25 Hz, 3 cycles per time-window, and frequency smoothing of 1 Hz was used.

### EEG data analysis

The main interest of the current study was to identify whether differences in processing temporal and numerical information could be observed. To facilitate EEG analysis, we only looked at standard trials, because standard trials always had the same duration and contained a fixed number of dots. To test for differences between the time and number condition in both the time and time–frequency domain, we created linear mixed-effect models (LMMs, lme4 package, version 1.1-10; Bates, Mächler, Bolker, & Walker, 2014) in R version 3.2.2 (R Development Core Team, 2008) entering amplitude averaged over the last 0.6 s before stimulus offset (1.2–1.8 s) and averaged over a central electrode cluster (FCz, C1, Cz, and C2) for each trial and participant as the dependent variable. We chose this particular time-window and channel selection based on the previous literature (e.g., Macar et al., 1999). Condition (*time*, coded as 0.5, or *numerosity*, coded as − 0.5) and position of standard (standard as S1, coded as − 0.5, or S2, coded as 0.5), as proposed by Bausenhart, Dyjas, & Ulrich (2015) and Dyjas, Bausenhart, & Ulrich (2012, 2014), were entered as predictors, while participant was entered as random intercept. We also tested more complex models incorporating information on the non-standard stimulus, but these models were not favored over the simpler models reported here.

For the analysis of time–frequency responses, the same model specifications were used, with the exception of the dependent variable. Here, we entered power averaged over the same time-window and electrode cluster as for the CNV analysis and for different frequency bands separately (delta: 2–4 Hz, theta: 4–8 Hz, alpha: 8–15 Hz, beta: 15–30 Hz).

In addition to this simple random-effects model, we also ran more complex random-effect models including random slopes for those fixed-effect factors that reached significance. As discussed by Bates et al. (2015), full random-effect models are often too complex to be accurately fitted by the data and do not converge, but including random effects for significant fixed effects does prevent spurious reporting of fixed effects. Whenever the more complex random-effects model is favored over the simple random-effects model, we report the complex random-effects models. Furthermore, for all fixed factors in the LMMs, we used Bayesian analyses to quantify the evidence in favor of the null hypothesis. To this end, we used the Bayesian Criterion Information (BIC) calculated for the model including the fixed factor and for the model without the factor as described in Wagenmakers (2007).

## Results

### Behavioral data

**Fig. 2:**
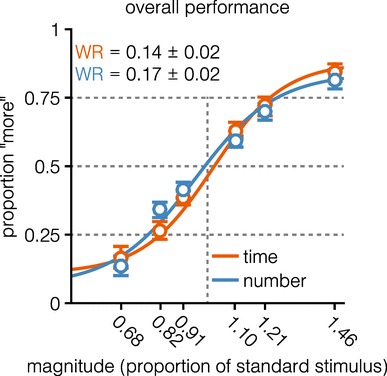
Behavioral performance. Psychometric curves and behavioral data depict overall performance in the time- and number-task. No statistically significant differences were found when comparing response accuracy (measured by the Weber Ratio, WR). For displaying purposes, psychometric curves were plotted using fitting parameters averaged across participants. Errors and error bars depict 95% confidence intervals

Following Lambrechts et al. (2013), we will focus on the Weber Ratio for all analyses, but analyses based on ‘proportion correct’ trials yielded the same pattern of results (for details, see osf.io/usjh4). Behavioral performance (Fig. 2) shows that response accuracy, measured by the Weber Ratio (*M*_time_ = 0.14, 95% CI [0.12, 0.16]; *M*_number_ = 0.17, 95% CI [0.15, 0.20]), was lower in the time-task (*t*(43) = − 2.28, *p* = .03, BF_01_ = 0.06 ± 0%), suggesting that the *number*-task was more difficult for participants than the time-task.

### Time-domain EEG signals

**Fig. 3:**
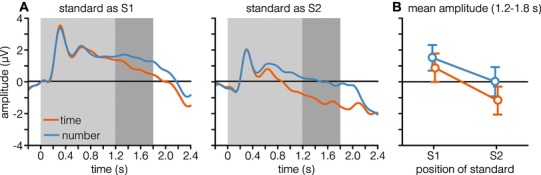
Time-domain signals. **A** Time courses of neural responses while processing the standard stimulus, averaged over central electrodes (FCz, C1, Cz, and C2) and plotted separately for both dimensions and positions of standard. Grey area marks the duration of stimulus presentation, while the dark grey area marks the time-window over which amplitude was averaged (**B**) and used for statistical analysis. **B** Amplitude averaged over the last 0.6 s of stimulus presentation (1.2–1.8 s). Data depicted in **B** were used for model analysis of CNV amplitude. Error bars depict 95% confidence intervals

Figure 3A shows the ERPs elicited by the standard stimulus occurring as S1 or S2 in the time and number condition. An overview of the results of the statistical analyses can be found in Table 1. Visual inspection of the ERP responses suggests that there is an overall higher onset ERP occurring 0.3–0.4 s after stimulus onset if the standard was presented as S1 compared to presentation as S2. LMM analysis of the CNV amplitude in the time-window spanning the last 0.6 s of stimulus presentation revealed that dimension influenced the magnitude of the CNV, with a more negative amplitude if the dimension is time and if the standard stimulus is presented as the second stimulus within a trial (visually depicted in Fig. 3B). Notably, no signs of CNV resolution (i.e., reversal of the negative trend of EEG signals after stimulus offset) can be seen in standard as S2 trials.

**Table 1:** Summary of LMM analyses’ results of fitting LMMs to predict CNV amplitude and power in different frequency bands

	Dimension			Position of standard				Dimension × position		
	Beta (SE)	*t*	BF_01_	Beta (SE)	*t*	BF_01_		Beta (SE)	*t*	BF_01_
Time-domain										
CNV amplitude	− 0.65^†^ (0.28)	− 2.32*	1	− 1.91^†^ (0.49)	− 3.88		0.18	0.64 (0.39)	0.39	30.05
Time**–**frequency domain										
Delta power	0.03 (0.07)	0.49	99.95	0.18^†^ (0.09)	2.01		16.36	− 0.29 (0.13)	− 2.17*	10.77
Theta power	0.08 (0.07)	1.03	66.02	0.10 (0.07)	1.40		42.21	− 0.30 (0.15)	− 2.01*	14.84
Alpha-power	− 0.04 (0.07)	− 0.52	98.28	− 0.47^†^ (0.10)	− 4.49***		0.03	0.14 (0.15)	0.97	70.17
Beta-power	− 0.02 (0.04)	− 0.48	100.31	− 0.39^†^ (0.06)	− 6.40***		< 0.01	− 0.03 (0.06)	− 0.46	101.16

Models included the predictors dimension (coded as − 0.5 for *number* and 0.5 for *time*), position of standard (coded as − 0.5 for S1 and 0.5 for S2), and their interaction

^†^Factor added as random slope, *p < .05, **p < .01, ***p < .001

### Time–frequency EEG responses

**Fig. 4:**
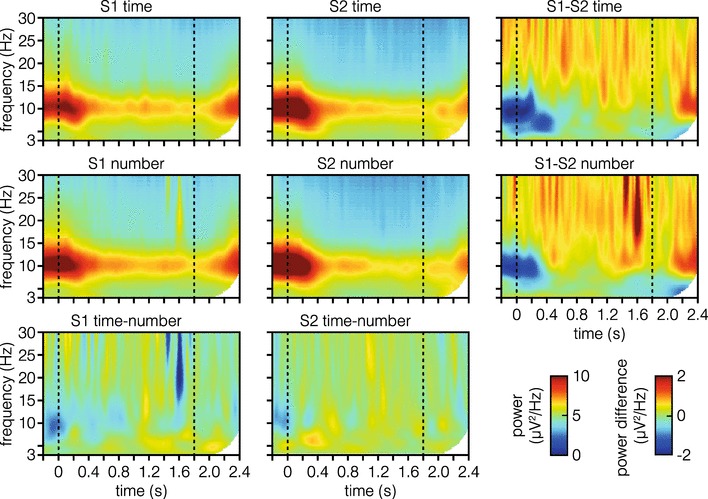
Time–frequency domain signals. Time–frequency domain signals averaged over central electrodes (FCz, C1, Cz, and C2) and plotted separately for both dimensions (first two rows) and positions of standard (first two columns). Data are not baseline corrected. Dashed lines mark stimulus onset and offset. The third column shows power difference between standard position S1 and S2. Likewise, the third row shows power differences between time and number dimension

Figure 4 visually summarizes the results of the time–frequency analysis. The same time-window as in the CNV analysis was used in LMM analyses testing whether power in specific frequency bands, including delta-, theta-, alpha-, and beta-bands, is modulated by dimension, position of standard or their interaction (summary of results can be found in Table 1). Results show that power in alpha- and beta-band is modulated by the position of the standard. Specifically, we found decreased alpha- and beta-power if the standard was presented as S2 (see Fig. 4, bottom row). No effects of dimension were found, and Bayes’ factors suggest that this is a convincing null result.

## Intermediate discussion

The current study aimed to investigate differences in EEG time and time–frequency domain signals between the processing of temporal and numerical information. We will save the discussion of our findings concerning the EEG data for the general discussion, and directly turn to the behavioral findings and their implications for the post hoc analyses described in the following section.

Typically, neuroimaging studies contrasting time with another dimension do not analyze behavioral data in great detail (but, see Coull et al., 2015). However, the subjectively perceived duration of a specific event is theorized to be influenced by other dimensions of the very same event (e.g., Walsh, 2003, 2014) in very similar task designs as the one employed in the current study. One well-studied example of how our subjective experience of time can be distorted is the effect of size on time in the visual domain: perceived duration increases as a function of increasing spatial magnitude, or, bigger stimuli are perceived as lasting longer (Cai, & Connell, 2016; Casasanto, & Boroditsky, 2008; Xuan, Zhang, He, & Chen, 2007). Another example is the effect of numerical magnitude on time perception: larger digit magnitudes during stimulus presentation lead to overestimated duration judgments (e.g., if the digits 9 and 2 are presented for the same interval on different trials; the interval corresponding to digit 9 will be overestimated) (Cai, & Wang, 2014; Oliveri et al., 2008). However, using different experimental paradigms or changing perceptual modality can change the direction of such interference effects. For example, Cai and Connell (2016) showed that when spatial information is presented to our haptic senses and time information via auditory channels, time affects spatial judgments, but not vice versa. Lambrechts, Walsh, and van Wassenhove (2013) found that when time, space, and number information are presented dynamically (i.e., perceptual evidence has to be accumulated over time in all three dimensions), duration judgments are resilient to spatial and numerical interferences, but time itself does influence judgments of the other two dimensions.

One way to experimentally test interactions between different dimensions is to manipulate congruency (e.g., Dormal, & Pesenti, 2013; Dormal, Seron, & Pesenti, 2006). For example, when experimental stimuli contain a time and space dimension, in a congruent trial, both dimensions vary in the same direction compared to a standard or comparison stimulus (e.g., longer and bigger). In an incongruent trial, the dimensions vary in opposite directions (e.g., shorter and bigger) and the target dimension is likely to be affected in the direction of the uncued condition (e.g., if *time* were the target dimension, the duration would likely be overestimated because of the influence of the dimension *space*). As congruency was also manipulated in the current study, the influence of the uncued condition could be assessed based on the behavioral responses.

Taken together, these behavioral findings indicate that participants might not only process information of the cued dimension, but also take into account information of the uncued dimension. Furthermore, direction and magnitude of congruency effects depend on the specific task design (e.g., which dimensions were used, whether information had to be accumulated or not) and might also differ between participants (i.e., some participants show stronger congruency effects than others). Especially, in neuroimaging studies in which a control task involving another dimension is simply subtracted from the time-task, either the paradigm needs to ensure that participants only use temporal information in the time condition and information of the control dimension in the control condition, or any observed neural differences should be weighted by the influence each of the dimensions has on the observed performance. As the nature of these tasks makes it practically impossible to ensure attention to just one dimension, it will be necessary to assess the relative usage of each of the dimensions when interpreting the neural signatures.

We conducted extensive post hoc analyses taking into account individual differences based on behavioral performance (i.e., congruency effects), and incorporated these results in the EEG analysis. In doing so, we can carefully disentangle the neural processing of temporal versus numerical information.

## Post hoc analyses

### Maximum-likelihood estimation (MLE) procedure

Because participants could potentially also use temporal information when judging number, and, respectively, numerical information when judging time, we used an MLE procedure to estimate, per participant, how each dimension was weighted in determining the response, separately for both task conditions. The model used the weighted sum of temporal and numerical evidence for each trial (*evidence*_total_, see Eq. 1); that is, parameter estimation was stimulus driven. Temporal (*evidence*_time_) and numerical evidence (*evidence*_number_) was determined by subtracting the magnitudes of the standard stimulus from the magnitudes of the non-standard stimulus and subsequently scaled from − 1 to 1 by dividing through maximal evidence possible (i.e., the more different the non-standard stimulus magnitudes were from the standard stimulus magnitudes, the more evidence). The weights *ω*_time_ and *ω*_number_ were estimated during the MLE procedure. *Evidence*_total_ was used to compute the probability of responses (shorter/fewer or longer/more) based on a standard normal distribution. The final weights were those that maximized the likelihood of the given series of responses over trials:

1 $${\text{evidenc}}{{\text{e}}_{{\text{total}}}}={\omega _{{\text{time}}}} \times {\text{evidenc}}{{\text{e}}_{{\text{time}}}}+{\omega _{{\text{number}}}} \times {\text{evidenc}}{{\text{e}}_{{\text{number}}}}.$$

Using this procedure, we obtained a weight for time and a weight for number for each task condition and participant. In an ideal case, assuming participants who completely follow instructions and ignore the irrelevant dimension, we find a high weight for time and a low weight for number if the task was to judge time, and the reversed pattern if the task was to judge numerosity.

**Fig. 5:**
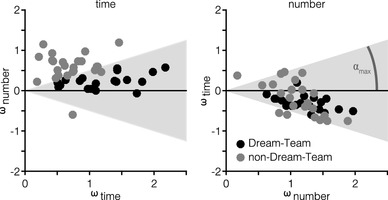
MLE output for the two task conditions: time and number. Each dot represents the estimated weights of one participant. Shaded grey area marks the selection criterion for Dream-Team membership, defined as cos^2^(*α*_max_) ≥ 0.8. For a participant to be grouped in the Dream-Team, their dot needs to fall into the grey area in both conditions

Figure 5 shows the *ω*-estimates for each participant in each condition. Participants showing no interference effects, for example, would have a high *ω*_time_ and a low *ω*_number_ in the time condition (i.e., their data would represent a dot at the positive end of the *x*-axis and close to the *x*-axis with regard to the *y*-component), and the reversed pattern in the number condition. Depending on the model output, we categorized participants into two groups: for a “Dream-Team”, we selected those participants who took, in both conditions, the relevant dimension (much) more strongly into account than the irrelevant dimension (see the shaded grey areas in Fig. 5, post hoc defined as cos^2^(*α*_max_) ≥ 0.8). Based on this selection criterion, we classified 20 participants as Dream-Team members and the remaining 24 participants as non-Dream-Team members. Differences in time- and number-weights between Dream-Team and non-Dream-Team membership are more pronounced in the time- than in the number-task. Bayesian two-sample *t* tests showed that differences are substantial if based on the time-task (*ω*_time_: BF_01_ = 0.01 ± 0%, *ω*_number_: BF_01_ = 0.01 ± 0%), but inconclusive if based on the number-task (*ω*_time_: BF_01_ = 2.00 ± 0.02%, *ω*_number_: BF_01_ = 2.35 ± 0.02%).

In the post hoc behavioral and EEG analysis, we analyzed data of the Dream-Team and non-Dream-Team groups separately.

### Behavioral congruency effects

Congruency effects are often tested by comparing the point of subjective equality, which can be calculated from individually fitted psychometric curves, across different conditions (e.g., Lambrechts et al., 2013). However, fitting individual psychometric curves for congruent and incongruent trials separately is problematic, because not all data points are available for every participant due to the stimulus sampling procedure employed in this study. Instead, we submitted the responses (“longer” = 1, “shorter” = 0) to a logistic generalized linear mixed-effect model in R. We entered a factor dimension (0.5 when participants were asked to pay attention to *time*, − 0.5 when attention was directed to *number*) and a factor encoding the position of standard (coded as − 0.5 if the standard appeared as S1, and as 0.5 if the standard appeared as S2) as fixed effects. In addition, we added the magnitude of the cued dimension (scaled from − 3, corresponding to T_1_/N_1_, to 3, corresponding to T_6_/N_6_), and the magnitude of the uncued dimension (same coding as for the cued magnitude) as fixed effects. Apart from testing the main effects of all entered factors, for both cued and uncued magnitude, we added the two-way interaction with dimension. Including this interaction allows for assessing the differential influence of each of the two different dimensions on the effect of the cued/uncued dimension on the recorded response. Participant was entered as random intercept.

As previously described, we report outcomes of more complex random-effects models if possible and if the more complex model is favored over the simple random-effects model. For all fixed factors in the LMM, we used Bayesian analyses to quantify the evidence in favor of the null hypothesis based on BIC, as described previously.

## Post hoc results

### Behavioral data

**Fig. 6:**
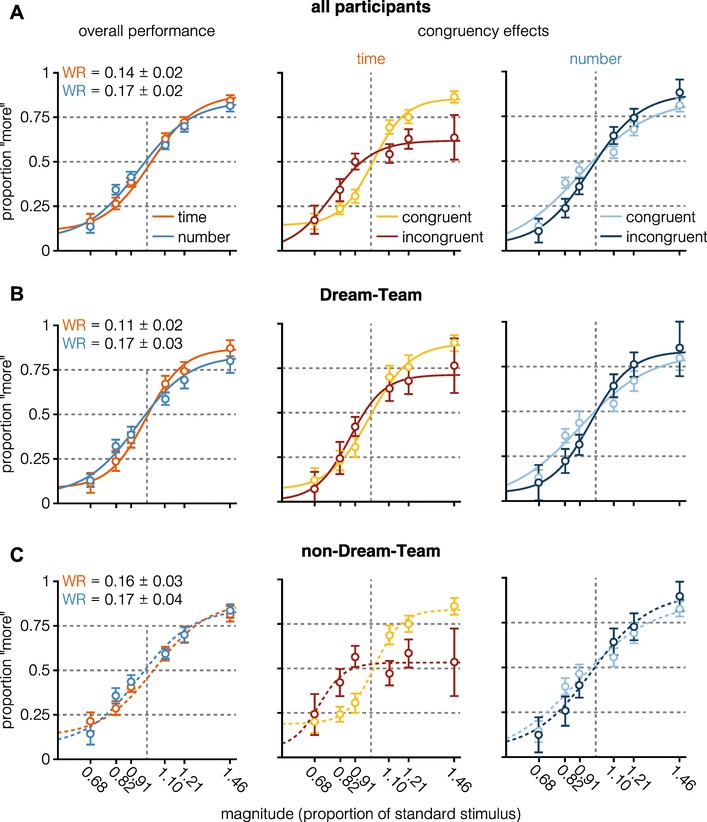
Overall **A**, Dream-Team **B**, and non-Dream-Team **C** behavioral performance. Parameters of psychometric fits depicting overall performance in the time- and number-task were averaged over participants. In none of the groups, statistically significant differences were found when comparing response accuracy (measured by the Weber Ratio, WR). Psychometric curves and behavioral data depict congruency effects for time and number separately. In a congruent trial, the magnitudes of the non-standard stimulus varied in the same direction (e.g., shorter duration and fewer dots than the standard stimulus), in an incongruent trial magnitudes varied in opposite directions, respectively (e.g., shorter duration and more dots than the standard). Psychometric curves depicting congruency effects for time and number were fitted using the pooled data of all participants within each group. Errors and error bars depict 95% confidence intervals

Behavioral performance (Fig. 6B, C, left column) shows that the Weber Ratio as a measure of response accuracy (Dream-Team: *M*_time_ = 0.11, 95% CI [0.09, 0.14]; *M*_number_ = 0.17, 95% CI [0.14, 0.20]; non-Dream-Team: *M*_time_ = 0.16, 95% CI [0.12, 0.19]; *M*_number_ = 0.17, 95% CI [0.14, 0.21]), did vary between tasks for Dream-Team participants, but not for non-Dream-Team participants (Dream-Team: BF_01_ = 0.34 ± 0%; non-Dream-Team: BF_01_ = 3.49 ± 0.04%).

Table 2 presents the results of LLM analyses on all participants combined (row 1, “all”), and for both Dream-Team (row 2, “DT”) and non-Dream-Team (Row 3, “nDT”). The LLM of all participants estimated the likelihood of a “more” response as a function of the entered predictors. The first column indicates that, for all (subsets of) participants, dimension did not influence the proportion of longer responses. The position of the standard stimulus (column 2) influences the responses for all (subsets of) participants, with the standard presented as the first stimulus increasing the likelihood of a longer response (i.e., for all participants, − 0.5 × − 0.46 = 0.23; cf. Bausenhart, Dyjas, & Ulrich 2015; Dyjas, Bausenhart, & Ulrich 2012, 2014). The magnitude of the cued dimension also influences the likelihood of a “more” response in all (subsets of) participants, demonstrating that participants, indeed, took into account the presented, cued magnitude. The last column also describes an effect that is similar for all (subsets of) participants, as, for all participants, the effect of the uncued dimension is conditional on which dimension was cued. If the cued dimension was time (coded as 0.5), the magnitude of the number dimension strengthens the effect of the cued dimension when congruent, demonstrating a strong congruency effect. However, when the dimension is number (coded as − 0.5), any main effects of congruency are diminished. This can be observed in Fig. 6, rightmost column, as congruency does not have a strong impact on “proportion more” in either set of participants. The middle column of Fig. 6 suggests stronger congruency effects for the non-Dream-Team than for the Dream-Team. This is reflected in Table 2, column 4 and 5, as the magnitude of the uncued dimension has a strong effect in the non-Dream-Team, and no effect (BF_01_ = 57.12) for the Dream-Team (column 4). The interaction between dimension and magnitude cued dimension strengthens this interpretation, as if the cued dimension is time, the Dream-Team takes the magnitude of the cued dimension even more into account (as the estimated beta is positive). On the other hand, the non-Dream-Team incorporates the time magnitude to a lesser extent, as their responses in the time-cued trials are driven to a larger degree by the numerosity dimension. Thus, Dream-Team members were more successful in ignoring the irrelevant information of the uncued dimension and based on their responses mainly on task-relevant information, as was the intention of the task.

**Table 2:** Summary of LMM analyses’ results of fitting LMMs to predict behavioral responses for all participants, as well as for participants in the Dream-Team (DT) and non-Dream-Team (nDT) separately

	Dimension			Position of standard			Magnitude cued dimension			Magnitude uncued dimension			Dimension × magnitude cued dimension			Dimension × magnitude uncued dimension		
	Beta (SE)	*z*	BF01	Beta (SE)	*z*	BF01	Beta (SE)	*z*	BF01	Beta (SE)	*z*	BF01	Beta (SE)	*z*	BF01	Beta (SE)	*z*	BF01
All	− 0.05 (0.04)	− 1.38	46.04	− 0.46 (0.04)	− 12.25***	< 0.01	4.19 (0.11)	39.04***	< 0.01	0.40 (0.09)	4.36***	< 0.01	− 0.77 (0.21)	− 3.61***	0.18	2.66 (0.18)	14.42***	< 0.01
DT	0.01 (0.06)	0.14	79.27	− 0.57 (0.06)	− 9.99***	< 0.01	4.95 (0.17)	29.52***	< 0.01	− 0.11 (0.14)	− 0.82	57.12	0.29 (0.33)	0.86	55.17	2.12 (0.28)	7.69***	< 0.01
nDT	− 0.10 (0.05)	− 2.03*	11.04	− 0.38 (0.05)	− 7.62***	< 0.01	3.63 (0.14)	25.82***	< 0.01	0.81 (0.13)	6.47***	< 0.01	− 1.52 (0.28)	− 5.40***	< 0.01	3.07 (0.25)	12.24***	< 0.01

Models included the predictors dimension (coded as − 0.5 for *number* and 0.5 for *time*), position of standard (coded as − 0.5 for S1 and 0.5 for S2), magnitude of the non-standard stimulus in the cued dimension (scaled from − 3, corresponding to T_1_/N_1_, to 3, corresponding to T_6_/N_6_), magnitude of the non-standard stimulus in the uncued dimension (same coding as for magnitude cued dimension), as well as two-way interactions of magnitude cued/uncued dimension and dimension. None of the full random-effects model did converge, and thus, we report results of the simple random-effects model

*p < .05, **p < .01, ***p < .001

### Time-domain EEG signals

**Fig. 7:**
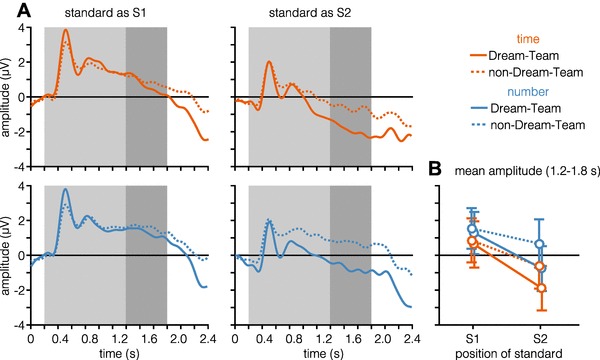
Time-domain signals for Dream-Team (solid lines) and non-Dream-Team (dashed lines) members. **A** Time courses of neural responses while processing the standard stimulus, averaged over central electrodes (FCz, C1, Cz, C2) and plotted separately for both dimensions, positions of standard, and Dream-Team membership. Grey area marks the duration of stimulus presentation, while the dark grey area marks the time-window over which amplitude was averaged (**B**) and used for statistical analysis. **B** Amplitude averaged over the last 0.6 s of stimulus presentation (1.2–1.8 s). Data depicted in **B** was used for post hoc model analysis of CNV amplitude. Error bars depict 95% confidence intervals

CNV time courses depicted in Fig. 7A show a trend towards an overall more negative CNV development in the Dream-Team compared to the non-Dream-Team. This trend is also visible in Fig. 7B, which shows CNV amplitude averaged over the last 0.6 s of stimulus presentation. Results of the model analysis conducted separately for Dream-Team and non-Dream-Team members are summarized in Table 3. The previously found significant influence of the factor dimension is also reflected in the analysis of both subsets of participants. However, the effect of the position of the standard stimulus was mainly driven by Dream-Team participants.

**Table 3:** Summary of LMM analyses’ results of fitting LMMs to predict CNV amplitude and power in different frequency bands in the Dream-Team (DT) and non-Dream-Team (nDT)

	Dimension			Position of standard				Dimension × position		
	Beta (SE)	*t*	BF01	Beta (SE)	*t*	BF01		Beta (SE)	*t*	BF01
Time-domain										
CNV amplitude	− 0.97^†^ (0.49)	− 4.93*	< 0.01	− 1.91^†^ (0.49)	− 3.88***		0.18	0.64 (0.39)	− 1.63	30.05
DT	− 0.95^†^ (0.30)	− 3.20**	1	− 2.45^†^ (0.55)	− 4.42***		0.08	− 0.51 (0.59)	− 0.87	52.19
nDT	− 0.97 (0.26)	− 3.73***	0.08	− 1.46^†^ (0.76)	− 1.91		15.19	− 0.74 (0.52)	− 1.41	30.73
Time–frequency domain										
Delta power	0.03 (0.07)	0.49	99.95	0.18^†^ (0.09)	2.01		16.36	− 0.29 (0.13)	− 2.17*	10.77
DT	0.01 (0.10)	0.14	75.21	0.09 (0.10)	0.93		49.34	− 0.26 (0.20)	− 1.31	32.15
nDT	0.05 (0.09)	0.52	72.65	0.24^†^ (0.13)	1.90		15.71	− 0.31 (0.18)	− 1.73	18.53
Theta power	0.08 (0.07)	1.03	66.02	0.10 (0.07)	1.40		42.21	− 0.30 (0.15)	− 2.01*	14.84
DT	0.25^†^ (0.10)	2.51*	3.55	0.25^†^ (0.11)	2.40*		4.82	− 0.23 (0.19)	− 1.17	38.47
nDT	− 0.07 (0.11)	− 0.62	68.87	− 0.02 (0.11)	− 0.16		82.20	− 0.36 (0.22)	− 1.67	20.62
Alpha-power	− 0.04 (0.07)	− 0.52	98.28	− 0.47^†^ (0.10)	− 4.49***		0.03	0.14 (0.15)	0.97	70.17
DT	0.11 (0.09)	1.23	35.62	− 0.29^†^ (0.09)	− 3.17**		1.10	0.10 (0.18)	0.56	64.86
nDT	− 0.16 (0.11)	− 1.43	29.80	− 0.62^†^ (0.17)	− 3.58**		0.46	0.18 (0.23)	0.79	61.12
Beta-power	− 0.02 (0.04)	− 0.48	100.31	− 0.39^†^ (0.06)	− 6.40***		< 0.01	− 0.03 (0.06)	− 0.46	101.16
DT	− 0.05 (0.05)	− 1.10	41.68	− 0.36^†^ (0.06)	− 5.79***		< 0.01	− 0.07 (0.09)	− 0.76	56.71
nDT	− 0.01 (0.03)	0.21	81.47	− 0.42^†^ (0.10)	− 4.23***		0.10	0.01 (0.07)	0.14	82.39

Models included the predictors dimension (coded as − 0.5 for *number* and 0.5 for *time*), position of standard (coded as − 0.5 for S1 and 0.5 for S2), and their interaction

^†^Factor added as random slope, *p < .05, **p < .01, ***p < .001

### Time–frequency EEG responses

**Fig. 8:**
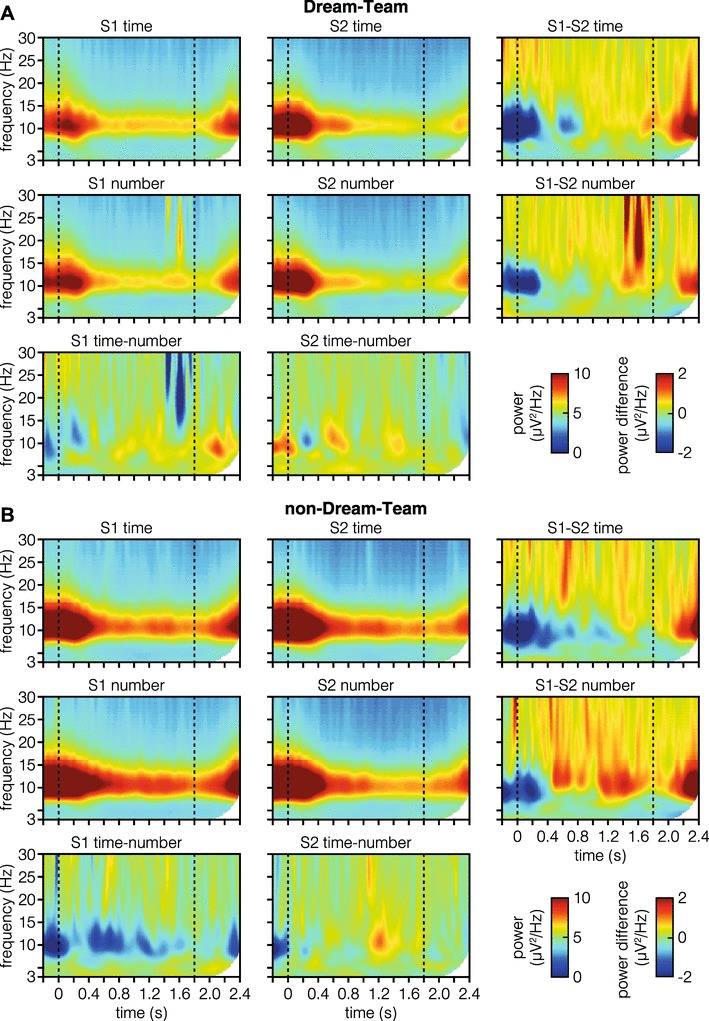
Time–frequency domain signals comparing Dream-Team and non-Dream-Team members. Data were averaged over central electrodes (FCz, C1, Cz, and C2) and plotted separately for both dimensions (first two rows) and positions of standard (first two columns) in **A** and **B**. Data are not baseline corrected. Dashed lines mark stimulus on- and offset. The third column shows power difference between standard position S1 and S2. Likewise, the third row shows power differences between time and number dimension

A summary of the time–frequency responses in the Dream- and non-Dream-Team can be seen in Fig. 8. Detailed results of the model analysis can be found in Table 3. The pattern of significant effects in the Dream-Team and non-Dream-Team subgroups is very similar to the one found in the previous analysis including all participants. Specifically, we again find that alpha- and beta-power decreases if the standard stimulus was presented as S2. Notably, when taking Bayes Factors into account, the effects found in the alpha-band seem to be less substantial in the Dream-Team compared to the non-Dream-Team.

Frequentist analyses suggest an effect for Dream-Team participants of dimension and position of standard in the theta-band; however, Bayes’ factors favor the null (i.e., there not being differences between dimensions and position of standard).

## Discussion

In the present study, an interval-timing task was compared to a dynamic numerosity-estimation task in search for EEG signatures that are unique to the processing of temporal information. Time-domain EEG results showed a CNV pattern in both the time- and numerosity-task that was significantly more negatively deflected in the time condition. However, as discussed below, a cautious interpretation of this phenomenon is required. No substantial differences between the processing of temporal and numerical information were found when looking at time–frequency domain signals. Even when selecting a subset of participants who more accurately followed instructions (i.e., those who mainly used temporal information in the timing task and numerical information in the number-task), the pattern of results did not change.

Figure 2 and the left-most column of Fig. 6 show the behavioral performance for both numerosity- and time-tasks. The difference in Weber Ratio between numerosity and time observed in the overall sample, with numerosity being more difficult than time, is driven by the Dream-Team sample, as no difference is observed when the performance of the non-Dream-Team participants is analyzed. This corroborates the finding that non-Dream-Team participants take numerosity into account in timing trials, even though this negatively affects their performance. We did not expect to observe this difference, as the parameters of the experimental design were based on pilot studies in which performance was non-distinguishable between conditions.

### Individual differences in magnitude of interference effects can inform EEG analysis

In neuroimaging studies, when looking for a comparison task for a timing task, the choice most often comes down to testing another dimension of the same stimulus used in the timing task (e.g., distance/time a dot travelled: Coull et al., 2015; prevalent color hue/duration of stimuli: Bueti, & Macaluso, 2011; Coull et al., 2004), because the two tasks are thought to be as similar as possible in terms of cognitive processes and demands. However, these tasks are used in behavioral experiments to study the magnitude interference effects, that is, how a task-irrelevant stimulus dimension influences the perceived magnitude of the task-relevant dimension (Walsh, 2003, 2014). Studies have typically yielded asymmetrical patterns of interference effects, with time estimation being affected by the magnitude of other dimensions, while time itself does not affect the perceived magnitude of other dimensions at all, or to the same extent (e.g., spatial magnitude/time: Cai, & Connell, 2016; Casasanto, & Boroditsky, 2008; Xuan et al., 2007; numerical magnitude/time: Cai, & Wang, 2014; Oliveri et al., 2008). If all stimulus dimensions are presented in an accumulative manner similar to time, time has been reported to be resilient to spatial and numerical interference (Lambrechts et al., 2013), a finding not supported by our results: in the current study, we found sizeable interference effects, and large individual differences in the magnitude of these interference effects, revealed by an MLE procedure estimating to which extent temporal and numerical evidence was taken into account. Although, in general, our data suggested that judgements on time were more likely to be affected by numerosity than the other way around, we identified a subset of participants based on the MLE estimates who showed little or no interference effects in either dimension (i.e., those participants seem to have selectively and rather exclusively used temporal information to make judgements on time and numerical information to make judgements on numerosity). The large inter-subject variability in interference effects could potentially also explain ambiguous findings about directionality of these effects (e.g., as reported in Lambrechts et al., 2013), in that they could, to a certain extent, be caused by participant sampling. Further, the direction of interference may depend on which and how many other dimensions are tested (e.g., integrating information of three dimensions as in Lambrechts, Walsh and van Wassenhove (2013) work might differ from integrating only two dimensions as in the current study), the exact paradigm (e.g., comparison, equality judgements, or reproduction tasks, as is discussed in Matthews, & Meck, 2016), and the nature of the task (e.g., whether the magnitude of the other dimension needs to be accumulated or not). More extensive research on the effect of task design on interference effects could potentially resolve these ambiguous findings.

Another insight gained from the MLE results is that, contrary to what was intended in this task, many participants did use task-irrelevant information of the uncued dimension when making a judgement on the cued dimension. This also means that, in these tasks, not only time but also numerosity is processed in the brain, which undermines the signal subtraction method employed in many fMRI studies and in the current EEG study. The subtraction method relies on the idea that the experimental and the control condition differ in the cognitive component of interest, while other cognitive processes remain equal (see, also for critique, Friston et al., 1996). The MLE procedure adopted in the current study presents one way to quantify behavioral interference effects on a single subject basis and inform subsequent EEG analysis. By selecting the Dream-Team participants based on the MLE estimates, we aimed to ensure to compare EEG signals during the processing of temporal information with signals recorded during the processing of numerical information.

The MLE estimates reflect the degree to which interference effects occurred on a single subject basis. However, care needs to be taken when interpreting these estimates, as the underlying mechanisms could, for example, reflect attentional or decision-making processes. Yet, we would argue in favor of the first interpretation: although the task-irrelevant dimension could, in principal, cause interference at a relatively late decision-making stage (Matthews, & Meck, 2016), recent research suggests that interference effects not only occur in comparison tasks where a decision is clearly needed, but also in temporal reproduction tasks which do not require to make a direct decision on stimulus properties (Chang, Tzeng, Hung, & Wu, 2011; Rammsayer, & Verner, 2014).

### Role of the CNV in timing

CNV patterns commonly observed in timing-tasks were apparent in both the time-task and the number-task, but they were more pronounced in the time-task. The latter result provides some support for the notion that CNV is intimately related to the accumulation of temporal information (Bendixen et al., 2005; Durstewitz, 2004; Macar, & Vidal, 2004; Macar et al., 1999; Pfeuty et al., 2005). Problematic for this notion, however, is the fact that the CNV differences between the two tasks were very similar for Dream-Team and non-Dream-Team participants. This is because as Dream-Team participants were found to rely more strongly and exclusively on temporal information in the time-task, a relatively more pronounced CNV development should have been expected for these participants in the time-task, resulting in a larger CNV difference between tasks, if CNV specifically reflects active accumulation of temporal evidence. Instead, rather than specifically tracking the accumulation of time (Kononowicz, & Penney, 2016; Ng, & Penney, 2014; van Rijn et al., 2011), these findings provide additional support to the notion that CNV reflects a time-critical but more general decision processes which might be best characterized as an accumulation process over time. This is plausible also in light of the nature of the task: by presenting the numerosity dimension dynamically, there is a clear temporal component not only in the time-, but also in the number-judgement task. Because the temporal component is task-critical in the time, but not in the number-task, this could explain our finding of a more pronounced CNV in the time-task. In fact, within the time perception literature, no other study has tested the CNV using a comparison task, although it is known that CNV patterns might also play an important role in non-timing evidence accumulation tasks (e.g., Boehm et al., 2014).

An interesting difference between the current study and other timing-CNV studies is that the stimuli marking an interval usually have distinct onset and offset (a circle changing its color). Here, interval onset and offset were fuzzier and less distinctly marked by the appearance and disappearance of only a few dots. This difference could explain why we did not see a clear CNV deflection at stimulus offset.

In the analysis as reported here, Dream-Team-ness was treated as a discrete grouping factor. However, when it was included in model analysis as a continuous measure per participant (see also Fig. 5), no qualitatively different results were observed (this additional analysis is available online at osf.io/usjh4).

### Role of neural oscillations in timing

While most of these studies have been largely exploratory, that is, they assessed whether there are any bands correlating with subjective temporal performance, other work has explored how oscillatory patterns could be related to timing at a theoretical level. For example, in a recent extension of the striatal beat frequency model (Buhusi, & Meck, 2005; Matell, & Meck, 2000, 2004), the integrative model for interval timing and working memory (Gu, van Rijn, & Meck, 2015) proposes that working memory and interval timing originate from the same underlying oscillatory processes. The model predicts that working memory is encoded in phase-amplitude coupled gamma–theta oscillations, while duration information is encoded in coupled theta–delta oscillations. However, there is no empirical evidence to support these predictions, including the current study.

Recent EEG studies have reported evidence, suggesting that beta oscillations may play an important role in timing, in that power of beta oscillations predicts behavioral performance (i.e., whether an interval was over- or underestimated in an interval production tasks, Kononowicz, & van Rijn, 2015). This finding has been replicated using MEG, with the addition that beta-power is greater in a temporal-judgement task as compared to a color-judgement task (Kulashekhar et al., 2016). In the current study, we did not find any differences between conditions in the beta-band. In fact, no frequency band tested here showed any substantial differences between the processing of temporal and numerical information, also when looking at Dream-Team participants exclusively.

The only convincing difference which we found when also taking Bayes Factors into account was a decrease in alpha and beta-power if the standard stimulus was presented as S2. This effect was very similar in Dream-Team and non-Dream-Team participants. These findings can be interpreted as being related to preparation processes. Alpha desynchronization has been connected to attentional processes (Klimesch, Sauseng, & Hanslmayr, 2007), so that lower alpha-power during the presentation of S2 could mean that participants were more attentive, because they also had to make a decision during that time. Beta desynchronization can be interpreted as an effect of motor or response preparation (Engel, & Fries, 2010; Zhang, Chen, Bressler, & Ding, 2008), given that S2 was closer to the required response in time and that in these trials, a decision could potentially be made immediately after the presentation of S1.

## Conclusion

We found that depending on the dimension, CNV patterns are negatively deflecting, with a more negative CNV if the task-critical dimension was time. However, a CNV pattern is also visible if the task-critical dimension was number. There are at least two possible conclusions for the CNV also to occur in the number-task: (1) the CNV reflects a timing process, and because of the nature of the task, we also have a temporal component in the number-task, or (2) the CNV reflects a time-critical process, but not the processing of temporal information per se. In our view, the specific pattern of results observed in this study provides stronger support for the latter view. Furthermore, we found no differences in EEG time–frequency signatures between the processing of temporal and dynamic numerical information; that is, the components and time-window which we examined are neither specific to the processing of time, nor to the processing of numerosity. There are at least three possible explanations: (1) an existing difference between the processing of temporal and numerical information in EEG data was not uncovered by our analyses (e.g., because on not addressing phase-amplitude coupling dynamics; see Gu et al., 2015). (2) There is no significant difference in our EEG data, because the relevant differences are either too subtle to be captured by mass-action techniques such as EEG, or, these magnitudes may be processed in other, non-cortical structures whose electric activity cannot easily be captured with EEG (e.g., time has been proposed to be encoded in cortico-basal ganglia-thalamic circuits; for a review, see Buhusi and Meck, 2005 or Gu, van Rijn and Meck, 2015). Or (3) there is only one mechanism or system for the processing of magnitudes, in general (as proposed in ATOM: *A General Theory Of Magnitude*, see Walsh, 2003, 2014).

An important issue that the current study addresses is that the outcome or findings of neuroimaging studies using these kinds of magnitude tasks could be misleading if it is not carefully assessed what participants actually did. The MLE procedure applied here proposes one way to capture individual differences in magnitude interference effects, and its outcome can inform analyses of neuroimaging data to link brain responses to actual behavior.

## Footnotes

1 This report contains two parts: in the Introduction we describe the original idea that inspired us to set up the experiment reported in this manuscript, and the first set of analyses and results. However, as expanded upon in the intermediate discussion, the results did not fully confirm the initial hypotheses. Explorations of the behavioral data suggested that an alternative view on the data might provide more insight, and in the second part of the manuscript we report an extensive post-hoc analysis of the EEG data conditional on behavioral performance. All experimental materials, analysis scripts and (preprocessed) data are available online on the Open Science Framework (osf.io/usjh4).

2 Durations were rounded to the second decimal (to ensure precise presentation timing) and number of dots was rounded to the nearest integer.
